# Effects of 12-Week Multivitamin and Omega-3 Supplementation on Micronutrient Levels and Red Blood Cell Fatty Acids in Pre-menopausal Women

**DOI:** 10.3389/fnut.2021.610382

**Published:** 2021-07-13

**Authors:** Shelby C. Osburn, Paul A. Roberson, Jessica A. Medler, Jacob Shake, Robert D. Arnold, Nima Alamdari, Luke R. Bucci, Arianne Vance, Mastaneh Sharafi, Kaelin C. Young, Michael D. Roberts

**Affiliations:** ^1^School of Kinesiology, Auburn University, Auburn, AL, United States; ^2^Harrison School of Pharmacy, Auburn University, Auburn, AL, United States; ^3^Ritual, Los Angeles, CA, United States; ^4^Cell Biology and Physiology, Edward via College of Osteopathic Medicine, Auburn, AL, United States

**Keywords:** multivitamin, women, omega-3, folate, vitamin D

## Abstract

The purpose of this study was to validate the efficacy of a customized vitamin-mineral supplement on blood biomarkers in pre-menopausal females. Women (21–40 years old) who were apparently healthy were recruited from the local community (ClinicalTrials.gov trial registration NCT03828097). Pretesting (PRE) occurred in the morning 5 ± 2 days following each participant's menses and involved a fasted blood draw, body mass assessment, and blood pressure assessment. Participants were then randomly assigned in a double-blinded fashion to either the multivitamins (MV) (*n* = 43) or placebo group (*n* = 51). Participants consumed two capsules per day with breakfast for 12 weeks. Following the trial, participants reported to the laboratory for POST assessments, which replicated PRE procedures. Red blood cell fatty acid and serum micronutrient analyses were performed in a blinded fashion at hematology laboratories. A group × time interaction was observed for serum vitamin D levels (*p* < 0.001). MV increased levels from PRE to POST (+43.7%, *p* < 0.001), whereas no change occurred in the placebo group. Additionally, 78% of MV participants at PRE exhibited inadequate vitamin D levels (<40 ng/dl), whereas only 30% exhibited levels below this threshold at POST. An interaction was also observed for serum folate levels (*p* < 0.001). MV increased serum folate from PRE to POST (*p* < 0.001), whereas no change occurred in the placebo group. Red blood cell omega-3 fatty acid content increased from PRE to POST in the MV group (*p* < 0.001) and placebo group (*p* < 0.05), although POST values were greater in the MV group (*p* < 0.001). An interaction was observed for serum HDL cholesterol levels (*p* = 0.047), and a non-significant increase in this variable from PRE to POST occurred in the MV group (*p* = 0.060). Four-day food recalls indicated MV increased intake of omega-3 fatty acids, vitamin D, folate, and other micronutrients. In summary, MV supplementation increased serum vitamin D, serum folate, and red blood cell omega-3 fatty acid levels. However, these data are limited to healthy females, and more research is needed to examine if MV can affect metabolic disturbances in individuals with micronutrient deficiencies.

## Introduction

Nutrient inadequacy is a public health concern in the U.S. According to recent evidence, 31% of the U.S. population may be at risk of at least one vitamin deficiency or anemia (1), with women among the most vulnerable groups. The recent report from “What We Eat in America- National Health and Nutrition Examination Survey (NHANES) 2013–2016” also indicates that several nutrients including vitamin D, vitamin E, and iron are underconsumed in American women's diets with the highest inadequacy prevalence found for vitamin D (dietary intake is below estimated average requirement for >97% of women ages 19–50) (2). Data from NHANES also suggest that the U.S. population's intake of long chain omega-3 fatty acids is limited leading to a high ratio of omega-6/omega-3 fatty acid intake (3). Epidemiological data suggest that this intake pattern promotes the pathogenesis of a number of chronic conditions [reviewed in (4)].

Due to the prevalence of nutrient inadequacy in the American diet, supplementation may be an appropriate strategy to help individuals meet latest recommendations. However, previous research has been limited to older adults, with little information available regarding healthy premenopausal females. Moreover, there is a debate regarding the efficacy of multivitamins as results from clinical studies have not been consistent (5–9). This inconsistency may be explained by the composition of products being tested that provide different nutrients, non-uniform forms of nutrients, and an array of dosages. For example, orally administered vitamin D3 has been shown to elevate total serum 25-hydroxy vitamin D levels (denoted as serum vitamin D throughout) more robustly than the D2 form (9). Omega-3 is a nutrient that is not typically found in multivitamins but has demonstrated efficacy related to cardiovascular health (10–12). While there is disparity in the composition and efficacy of products tested, there is also the possibility of product label claims not meeting their actual analytic content when tested (13). Further limitations and inconsistencies may also be explained by the different types of studies being conducted (e.g., observational studies vs. clinical trials), as well as the demographic characteristics of subjects. Therefore, the primary purpose of this study was to conduct a clinical trial to validate the efficacy of a multivitamin and omega-3 supplement (termed MV herein), and determine whether 12 weeks of supplementation affected select serum micronutrient levels and red blood cell omega-3 fatty acid profiles in pre-menopausal women. We hypothesized that MV supplementation would increase serum vitamin D and folate levels compared to placebo supplementation. Moreover, we hypothesized that MV supplementation would increase red blood cell fatty acid levels compared to placebo supplementation.

## Methods

### Ethics Approval and Participant Inclusion Criteria

All procedures in this study were approved by the Institutional Review Board at Auburn University (Protocol # 18-092 MR 1803), and this study conformed to the standards set by the latest revision of the Declaration of Helsinki. This study was also registered prior to data collection at clinicaltrials.gov (ClinicalTrials.gov trial registration NCT03828097). Potential participants were recruited through fliers from the local Auburn and Opelika communities. Eligible participants had to be female between the ages of 21–40 and free of cardio-metabolic diseases (e.g., morbid obesity, type II diabetes, severe hypertension). Participants had to self-report having a regular menstrual cycle and could not be former or current smokers. If participants consumed multi-vitamins, they had to discontinue usage 1 month prior to the study. Additionally, participants were screened via questionnaire to ensure that they had normal dietary habits, and were not consuming other nutritional supplements or medications which would affect serum micronutrients. Interested participants provided verbal and written consent to participate prior to data collection procedures outlined below.

### CONSORT Diagram and Characteristics of Participants

**Figure 1:**
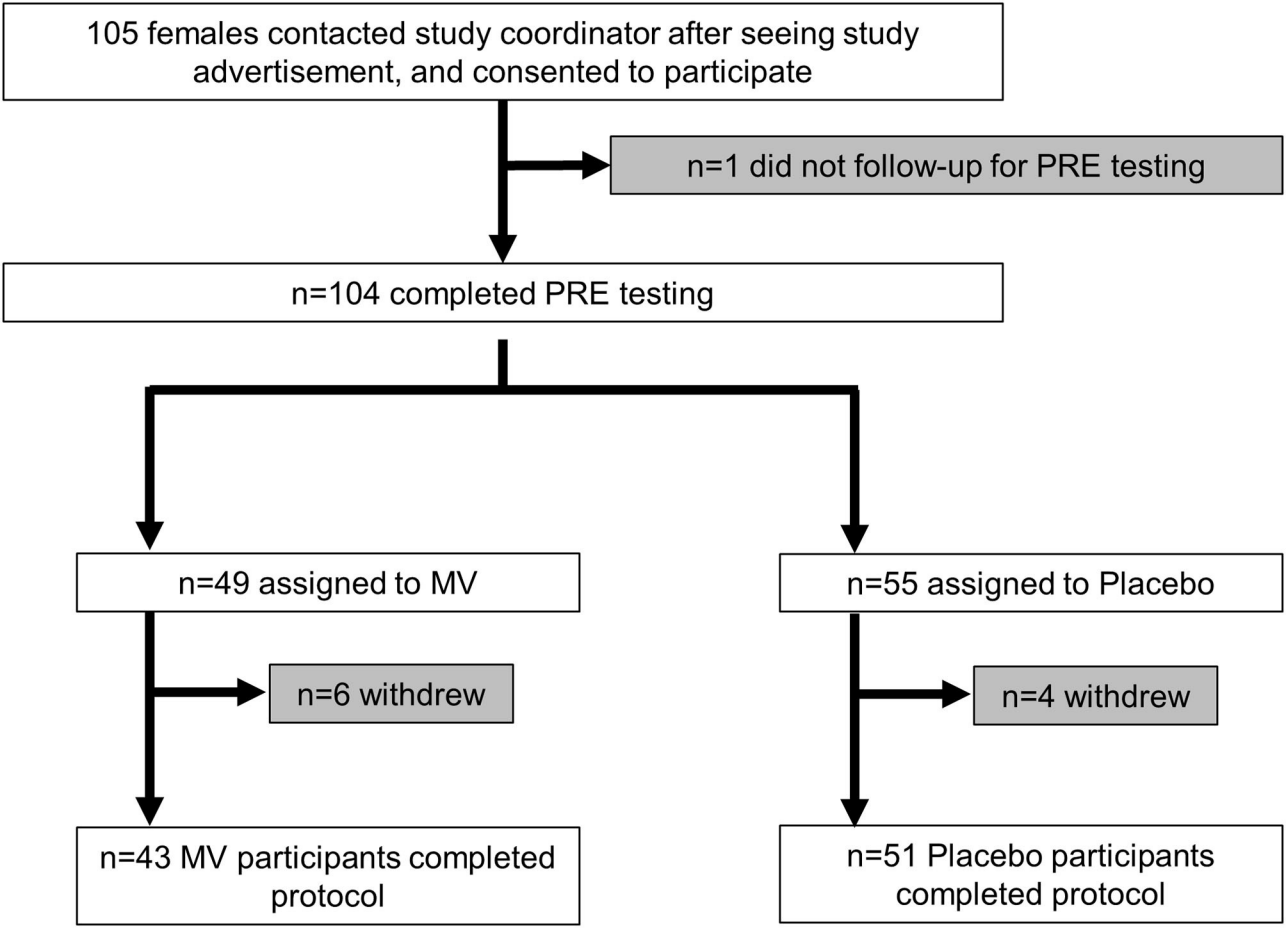
CONSORT diagram. This figure illustrates the number of participants from enrollment to completion of the trial.

The CONSORT diagram of the study is presented in Figure 1. In short, 105 females were deemed eligible and consented to partake in the study. One participant did not return following the consent visit to partake in pretesting. Of the 104 participants that engaged in PRE testing and were randomized to the MV or placebo group, 10 participants removed their self from the study during the 12-week trial due to personal reasons, but none discontinued due to adverse events. Thus, a total of 94 participants (*n* = 43 MV, *n* = 51 placebo) completed the study.

### Testing Sessions

The testing session detailed below occurred during the morning hours (06:00–09:00) following an overnight fast, and procedures are described in the order that they occurred. These sessions occurred prior to the 12-week supplement trial (PRE), and after supplementation (POST). Notably, menstrual cycle logs were given to participants, and we requested that participants report to PRE and POST testing sessions 5 ± 2 days following each participant's menses of their last menstrual cycle. Participants were also given 4-day food logs prior to the PRE and POST testing sessions, and we requested that they return these logs during the visits after having completed the log over two typical weekdays and weekend days. Food log analyses are discussed later in the methods.

### Urine Testing and Body Mass Assessment

At the beginning of each testing session, each participant performed a urine pregnancy test to confirm that she was not pregnant. Following this test, body mass and height were obtained using a digital column scale (Seca 769; Hanover, MD, USA) with weights and heights being collected to the nearest 0.1 kg and 0.5 cm, respectively.

### Resting Heart Rate and Blood Pressure Assessments

Following body mass assessment participants sat in a chair for 5 min to obtain resting heart rate, systolic, and diastolic blood pressure. Measurements were obtained at the brachial artery using an automated blood pressure cuff (model BP785N; Omron Healthcare, INC., Lake Forest, IL, USA).

### Blood Collection and Analyses

Venous blood samples were collected from the arm into a 6 ml serum separator tube (BD Vacutainer; Franklin Lakes, NJ, USA) and 4 ml EDTA tube (BD Vacutainer) using standard aseptic phlebotomy techniques. Serum tubes were centrifuged at 3,500 g for 10 min at room temperature. Serum aliquots were then placed in 3 ml polypropylene tubes and stored at −80°C until shipment for downstream assays. Whole blood from EDTA tubes was aliquoted into 1.7 ml polypropylene tubes and stored at −80°C until shipment for red blood cell fatty acid analysis.

Following completion of specimen collection, frozen whole blood was shipped on dry ice to a commercial entity (OmegaQuant; Sioux Falls, SD, USA) who performed red blood cell fatty acid composition analysis in a central CLIA-certified laboratory. Serum tubes were sent to the local Auburn-Opelika hospital and analyzed for general chem-20 panels as previously reported by our laboratory (14, 15). Serum was also analyzed for vitamin D 25-OH levels and folate levels. Critically, all tubes had no participant identifier information on them except a subject number (S1 through S105) and the term “PRE” or “POST” to indicate the time point when blood was obtained.

### Supplementation Period and Participant Monitoring

Following IRB approval, the manufacturer shipped 100 MV (Natals, Inc, known as Ritual^TM^) bottles and 100 placebo bottles to Auburn investigators. Each bottle contained 200 pills, and pills were visually-identical and similarly-flavored between conditions. Each serving of MV (per two beadlets-in-capsule serving) contained 50 μg (2,000 IU) of vitamin D3, 6.7 mg alpha tocopherol (10 IU) of vitamin E as mixed tocopherols, 90 μg vitamin K2 MK7, 600 μg (1,000 DFEs) 5-methyltetrahydrofolate, 8 μg B12, 8 mg iron, 50 mg magnesium, 1 mg boron, and 320 mg omega-3 fatty acids. Each serving of placebo contained predominantly safflower with cellulose beadlets. The only information on each bottle was a random five-digit code generated by the manufacturer. Auburn investigators distributed one bottle to each participant in a double-blinded fashion following the blood collection during PRE, and briefed participants on the supplementation protocol. In short, participants were instructed to consume two capsules per day of their respective supplement with breakfast for 12 weeks. Participants were also instructed to start supplementation the day following PRE testing, end supplementation the day prior to POST testing, and return bottles with unconsumed pills at the POST testing session. During the supplementation period, the study coordinator emailed participants on a monthly basis to monitor supplement compliance and side effects. Following the procurement of all blood data, the manufacturer debriefed Auburn researchers by providing a key containing information relating the 5-digit bottle identification codes to the MV or placebo capsules.

Notably, MV was formulated with the intent to provide additional support to the female diet based on data from the National Health and Nutrition Examination Survey suggesting that intakes of the micronutrients contained within the supplement, as well as omega-3 fatty acid intakes, are low (www.usda.gov). Micronutrient and omega-3 quantities were validated for MV using high performance liquid chromatography and Inductively Coupled Plasma method by a third party (Eurofins Scientific, Petaluma, CA, USA).

### Food Log Analysis

Pre- and post-trial 4-day food logs were analyzed using commercially available software (ESHA Food Processor v.11.1, ESHA Research; Salem, OR, USA). All data are presented as daily intake values, which were averaged over the 4-day entries. For the MV group, micronutrient values were analyzed with and without the MV contents and compared to the placebo group.

### Statistics

Prior to data collection, sample size calculations were performed using prior research demonstrating that supplements containing vitamin D and/or folate could significantly elevate blood levels of these micronutrients. We determined that an *n*-size of ~30–40 individuals per group would yield adequate power to detect significant differences in these variables.

Statistical analyses were performed in SPSS (Version 25; IBM SPSS Statistics Software, Chicago, IL, USA). Prior to statistical testing, outlier removal was performed on main outcome variables by removing participant data points with values that exceeded ±3 standard deviations from the mean in each group at PRE and/or POST. Notably, outlier removal was decided upon *a priori*. If a participant was removed at PRE, their data was also removed at post and vice versa. Thereafter, dependent variables at PRE and POST were analyzed for normality using Shapiro-Wilk tests. Select dependent variables at baseline were analyzed between groups using independent samples *t*-tests, and all other dependent variables were analyzed between groups over time using two-way (group or supplement condition × time) repeated measures ANOVAs. Results for each dependent variable are presented as main effects of time and group, and a group × time interaction. When a significant group × time interaction was observed, LSD *post-hoc* tests were performed within and between groups at the PRE and POST time points to determine where differences occurred. Details regarding outlier removal, data normality, and parametric vs. non-parametric *post-hoc* tests for each dependent variable are detailed in the results section. All data in tables and figures are presented as mean ± standard deviation values, and statistical significance was set at *p* < 0.05.

## Results

### Participant Characteristics

Participants in the placebo group were 24 ± 4 years old and had a body mass index (BMI) of 23.3 ± 2.9 kg/m^2^. Participants in the MV group were 23 ± 3 years old and had a BMI of 23.1 ± 2.7 kg/m^2^. There were no differences between groups for these variables at baseline (age *p* = 0.584, BMI *p* = 0.632).

### Self-Reported Side Effects and Compliance

Self-reported side effects data during the 12-week trial are presented in Table 1. In short, seven MV participants reported minor adverse events, and six placebo participants reported minor adverse events. Notably, one participant in each group a side effect that persisted throughout the study (i.e., dry skin, and acne).

**Table 1:** Perceived side effects.

**Group**	**Perceived side effect**
**MV GROUP**	
Participant 1	“Bit of an upset stomach” (reported at month 2)
Participant 2	“Indigestion” (reported at month 1)
Participant 3	“Increased acne, dry skin, dry hair”
	(reported at months 1 and 2)
Participant 4	“Occasional digestive upset” (reported at month 2)
Participant 5	“Brittle nails” (reported at month 1)
Participant 6	“Weaker nails” (reported at month 2)
Participant 7	“Headaches” (reported at month 1),
	“Breakouts” (reported at month 2)
**PLACEBO GROUP**	
Participant 1	“Hair loss” (reported at month 2)
Participant 2	“More breakouts” (reported at month 2)
Participant 3	“Dry skin, acne” (reported at month 2)
Participant 4	“Dry skin around the mouth, acne, red cheeks”
	(reported at months 1 and 2)
Participant 5	“Occasional upset stomach” (reported at month 1)
Participant 6	“More breakouts” (reported at month 2)

*Perceived side effects from email and verbal communication during the 12-week supplementation period*.

Regarding supplementation compliance, 48/51 of the placebo participants and 42/43 MV participants returned bottles with remaining pills at the end of the study. Over the duration of the trial participants were expected to consume 168 pills (12 weeks × 7 days per week × 2 pills per day). However, this varied between individuals given differences in menstrual lengths in proximity to POST testing. Of the participants that returned bottles, the MV group consumed 164 ± 9 pills during the study, and the placebo group consumed 167 ± 9 pills during the study (*p* = 0.105, data not shown). The three participants that did not return pill bottles verbally confirmed that they were compliant throughout the study.

### Pre- and Post-trial Body Mass, Heart Rate, and Blood Pressure

Body mass, heart rate, and blood pressure data at PRE and POST are presented in Table 2. No significant group × time interactions were evident for any of these variables.

**Table 2:** Body mass, heart rate, and blood pressure.

**Variable**	**Time**	**Placebo**	**MV**	**Statistics**
		**(*n* = 51)**	**(*n* = 43)**	
Body mass (kg)	PRE	63.8 ± 9.3	64.5 ± 9.0	Time *p* = 0.041
	POST	64.4 ± 9.7	64.6 ± 9.2	G × T *p* = 0.192
Heart rate (beats per minute)	PRE	67 ± 10	65 ± 9	Time *p* = 0.987
	POST	68 ± 12	68 ± 12	G × T *p* = 0.579
Systolic blood pressure (mm Hg)	PRE	110 ± 9	108 ± 8	Time *p* = 0.378
	POST	109 ± 9	108 ± 8	G × T *p* = 0.885
Diastolic blood pressure (mm Hg)	PRE	72 ± 7	73 ± 7	Time *p* = 0.077
	POST	74 ± 8	73 ± 7	G × T *p* = 0.323

*Pre- and post-trial body mass, heart rate and blood pressure data. All data presented as mean ± standard deviation values. No significant group × time (G × T) interactions were evident*.

### Pre- and Post-trial Vitamin D 25-OH and Folate Levels

**Figure 2:**
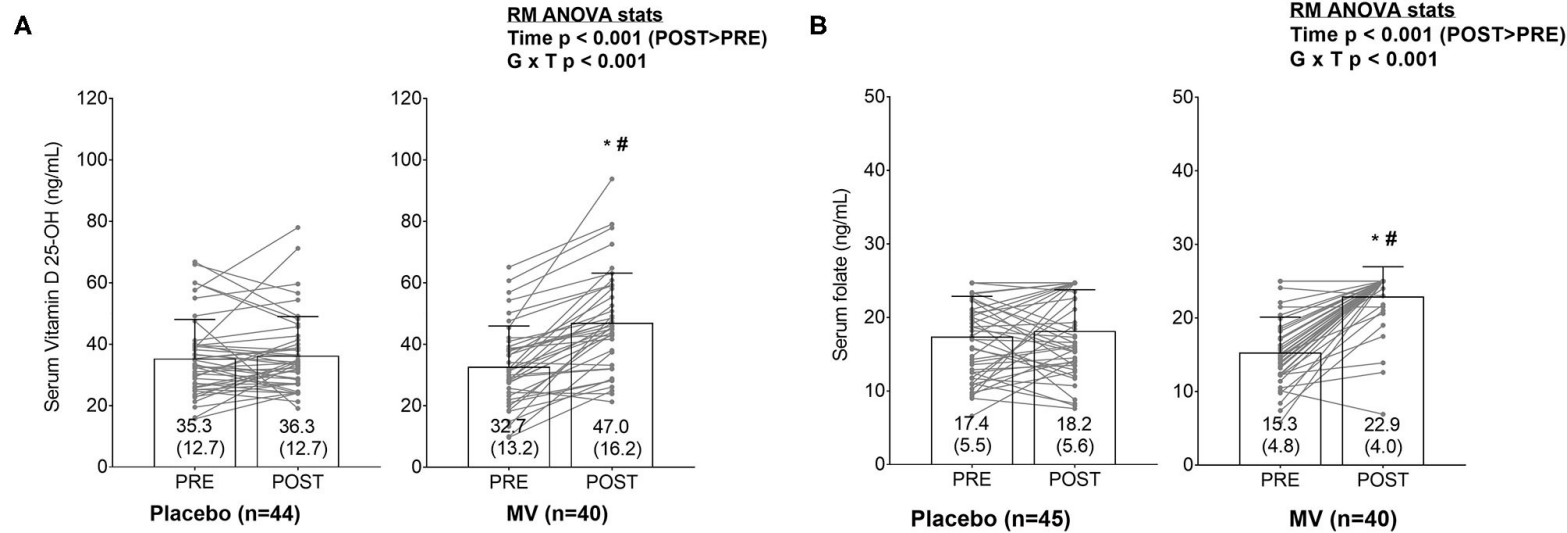
**(A,B)** Changes in serum vitamin D and folate levels between groups. Bar graphs are plotted as means ± standard deviation values (which are also indicated numerically at the bottom of each bar), and gray data points indicate individual respondents. The significant group × time (G × T) interactions for each variable prompted LSD *post-hoc* tests within each group and between groups at each time point. Both serum markers increased from PRE to POST in MV group (*p < 0.05). Additionally, POST values for both serum markers were elevated in the MV vs. placebo group (#*p* < 0.05).

Of the 43 MV and 51 placebo participants that completed the study only 40 and 45, respectively, yielded enough serum for vitamin D 25-OH and folate analyses. At PRE and POST, one participant in the placebo group displayed vitamin D values that were >3 standard deviations greater than the group mean at PRE and POST; thus, this participant was removed from analyses. Following the removal of this participant, serum vitamin D values at PRE and POST were not normally distributed (Shapiro-Wilk *p* < 0.001). Two-way ANOVAs indicated a group × time interaction existed for serum vitamin D (*p* < 0.001, Figure 2A). Parametric *post-hoc* analyses indicated this variable increased from PRE to POST in the MV group (*p* < 0.001). Additionally, parametric *post-hoc* analysis indicated POST values for serum vitamin D was greater in the MV vs. placebo group (*p* = 0.001). Non-parametric *post-hoc* analyses yielded similar findings. The current paper considered serum vitamin D levels below 40 ng/dl threshold to be suboptimal (16, 17) and the result showed that at PRE, 34/45 (76%) placebo participants and 31/40 (78%) MV participants had values below this threshold. At post, however, only 12/40 (30%) MV participants were still below this threshold, whereas 32/45 (71%) of placebo participants were below this threshold.

Although there were no outliers for serum folate, values were also non-normally distributed at PRE and POST. Additionally, the upper assay limit for serum folate was 24.8 ng/ml. Some of the placebo (*n* = 6) and several MV participants (*n* = 2) realized this threshold at PRE, and some realized this threshold at POST as well (placebo *n* = 13, MV *n* = 33). Two-way ANOVAs indicated a group × time interaction existed for serum folate (*p* < 0.001, Figure 2B). Parametric *post-hoc* analyses indicated this variable increased from PRE to POST in the MV group (*p* < 0.001). Additionally, parametric *post-hoc* analysis indicated POST values for serum folate was greater in the MV vs. placebo group (*p* < 0.001). Non-parametric *post-hoc* analyses yielded similar findings.

### Red Blood Cell Fatty Acid Content

Select red blood cell fatty acid concentrations are presented in Table 3, and all data are presented as the percent contribution of each fatty acid to the total fatty acid pool. Of the 43 MV participants that completed the study, only 42 yielded enough blood for analyses. There were no outliers for omega-3 fatty acids or linoleic acid. There was one outlier in the placebo group for docosahexaenoic acid (DHA) and eicosapentaenoic acid (EPA). There was an additional outlier in the placebo group for arachidonic acid, and one outlier in the MV group for this variable as well. When removing these outlier values, all variables except for EPA were normally distributed. Two-way ANOVAs indicated group × time interactions existed for omega-3 fatty acids (*p* < 0.001), DHA (*p* < 0.001), EPA (*p* < 0.001), arachidonic acid (*p* < 0.001), and the omega-6/omega-3 ratio (*p* < 0.001). *Post-hoc* analyses indicated an increase from PRE to POST in omega-3 fatty acids and DHA occurred in the placebo group (*p* < 0.05), and increases in omega-3 fatty acids, DHA, and EPA occurred in the MV group (*p* < 0.001). Additionally, the omega-6/omega-3 ratio decreased in the MV group (*p* < 0.001), whereas no change occurred in the placebo group. POST values for omega-3 fatty acids, DHA, and EPA were greater in the MV vs. placebo group (*p* < 0.001). Additionally, arachidonic acid and the omega-6/omega-3 ratio decreased in the MV group from PRE to POST (*p* < 0.05 and *p* < 0.001, respectively). Arachidonic acid values also increased in the placebo group from PRE to POST (*p* = 0.001), and values in the placebo group at POST were greater than the MV group (*p* = 0.001). Non-parametric *post-hoc* analyses yielded similar findings for EPA; specifically, a Wilcoxin signed rank test indicated this variable increased from PRE to POST in the MV group (*p* < 0.001), and a Mann-Whitney U test indicated values for this variable were greater in the MV group at POST (*p* < 0.001).

**Table 3:** Pre- and post-trial red blood cell fatty acid data.

**Variable (units)**	**Time**	**Placebo**	**MV**	**Statistics**
		**(*n* = 49–51)**	**(*n* = 41–42)**	
Omega-3 (ω-3) fatty acids (%)	PRE	3.5 ± 1.2	3.2 ± 0.5	Time *p* < 0.001
	POST	3.7 ± 1.3^*^	4.7 ± 0.7^*^^,^^#^	G × T *p* < 0.001
DHA (22:6), ω-3 (%)	PRE	2.9 ± 0.7	2.9 ± 0.5	Time *p* < 0.001
	POST	3.1 ± 0.8^*^	4.1 ± 0.6^*^^,^^#^	G × T *p* < 0.001
EPA (20:5), ω-3 (%)	PRE	0.4 ± 0.2	0.4 ± 0.1	Time *p* < 0.001
	POST	0.4 ± 0.2	0.6 ± 0.2^*^^,^^#^	G × T *p* < 0.001
Linoleic (18:2), ω-6 (%)	PRE	20.0 ± 2.2	20.2 ± 1.8	Time *p* = 0.003
	POST	19.1 ± 2.8	19.7 ± 2.3	G × T *p* = 0.316
Arachidonic (20:4), ω-6 (%)	PRE	12.5 ± 1.2	12.4 ± 1.4	Time *p* = 0.304
	POST	13.0 ± 1.5^*^^,^^#^	12.1 ± 1.3^†^	G × T *p* < 0.001
ω-6/ω-3 (ratio)	PRE	7.8 ± 1.5	7.8 ± 1.1	Time *p* < 0.001
	POST	7.6 ± 1.5	5.8 ± 1.0^*^^,^^#^	G × T *p* < 0.001

Data are presented as mean ± SD values. Two-way ANOVAs indicated group × time (G × T) interactions existed for all variables but linoleic acid. Several variables increased within the placebo and/or MV groups from PRE to POST (*p < 0.05), and POST values in some of these variables were different in the MV vs. placebo group (#p < 0.05). Notably, the significant decrease in red blood cell arachidonic acid content is indicated by “†”.

### Pre- and Post-trial Serum Markers of Clinical Safety

Serum markers of clinical safety are presented in Table 4 below. No outliers were detected for any of these variables in either group. Only serum phosphorus and all cholesterol-related variables were normally distributed. A two-way ANOVA indicated a group × time interaction existed for serum high-density lipoprotein (HDL) cholesterol. However, *post-hoc* analyses indicated a non-significant increase in this marker occurred in the MV group (*p* = 0.061), whereas no change occurred in the placebo group (*p* > 0.10). No other group × time interactions existed for other assayed variables. Given that HDL cholesterol was the only variable to show a group × time interaction, and this variable was normally distributed, non-parametric *post-hoc* statistics were not performed.

**Table 4:** Serum markers of clinical safety.

**Variable (units)**	**Time**	**Placebo (*n* = 51)**	**MV (*n* = 43)**	**Statistics**
Serum glucose (mg/dl)	PRE	86.6 ± 6.4	87.4 ± 9.4	Time *p* = 0.006
	POST	83.2 ± 7.3	86.3 ± 7.9	G × T *p* = 0.159
Serum sodium (mM)	PRE	137 ± 5	139 ± 9	Time *p* = 0.008
	POST	138 ± 4	140 ± 10	G × T *p* = 0.859
Serum potassium (mM)	PRE	4.0 ± 0.5	3.9 ± 0.3	Time *p* = 0.004
	POST	3.9 ± 0.3	3.8 ± 0.3	G × T *p* = 0.565
Serum chloride (mM)	PRE	103 ± 3	103 ± 5	Time *p* = 0.743
	POST	103 ± 4	103 ± 5	G × T *p* = 0.865
Serum calcium (mg/dl)	PRE	9.0 ± 0.5	8.9 ± 0.8	Time *p* = 0.865
	POST	9.0 ± 0.6	8.9 ± 0.8	G × T *p* = 0.974
Serum phosphorous (mg/dl)	PRE	3.6 ± 0.4	3.6 ± 0.4	Time *p* = 0.038
	POST	3.7 ± 0.4	3.7 ± 0.4	G × T *p* = 0.825
Blood urea nitrogen (mg/dl)	PRE	11.5 ± 3.0	11.5 ± 2.6	Time *p* = 0.620
	POST	11.3 ± 2.6	11.5 ± 2.7	G × T *p* = 0.620
Serum creatinine (mg/dl)	PRE	0.8 ± 0.1	0.8 ± 0.1	Time *p* = 0.378
	POST	0.8 ± 0.1	0.8 ± 0.1	G × T *p* = 0.378
Calculated GFR (mL/min/1.73 m^2^)	PRE	71.3 ± 10.8	73.5 ± 13.2	Time *p* = 0.600
	POST	71.9 ± 11.9	72.0 ± 12.4	G × T *p* = 0.193
Serum albumin (mg/dl)	PRE	4.2 ± 0.3	4.2 ± 0.4	Time *p* = 0.301
	POST	4.2 ± 0.3	4.1 ± 0.3	G × T *p* = 0.936
Serum total cholesterol (mg/dl)	PRE	165.7 ± 25.9	152.5 ± 27.9	Time *p* = 0.735
	POST	162.4 ± 30.9	154.4 ± 26.3	G × T *p* = 0.211
Serum HDL (mg/dl)	PRE	58.5 ± 9.6	52.7 ± 10.4	Time *p* = 0.441
	POST	57.6 ± 9.9	54.9 ± 10.9	G × T *p* = 0.047
Serum LDL (mg/dl)	PRE	93.9 ± 23.4	89.0 ± 26.1	Time *p* = 0.919
	POST	93.5 ± 26.1	89.0 ± 24.4	G × T *p* = 0.907
Serum triglycerides (mg/dl)	PRE	82.7 ± 29.5	83.7 ± 40.6	Time *p* = 0.375
	POST	83.4 ± 43.3	77.1 ± 28.4	G × T *p* = 0.217

*All data presented as mean ± standard deviation values. The serum HDL G × T interaction was due to the non-significant increase in this variable in the MV group from PRE to POST (p = 0.061). No other G × T interactions were evident*.

### Pre- and Post-trial Self-Reported Food Log Data

Self-reported food log data are presented in Table 5 below, and these data in the MV group contain the omega-3 and micronutrient values added from daily MV supplementation. Of the 43 MV and 51 placebo participants that completed the study, only 41 and 46, respectively, turned in food logs at both the PRE and POST time points. None of these variables were normally distributed. Two-way ANOVAs indicated group × time interactions existed for omega-3 fat intake (*p* = 0.008), folate intake (*p* < 0.001), iron intake (*p* < 0.001), vitamin B12 intake (*p* < 0.001), vitamin D intake (*p* < 0.001), vitamin E intake (*p* < 0.001), and vitamin K intake (*p* < 0.009). Parametric *post-hoc* analyses indicated that all of these variables increased from PRE to POST in the MV group only (*p* < 0.05), and non-parametric *post-hoc* analyses yielded similar results. Additionally, with the exception of omega-3 intake, self-reported intakes for all of the other aforementioned variables were greater at POST in the MV vs. placebo group (*p* < 0.05), and non-parametric *post-hoc* analyses yielded similar results. There was an interaction for omega-6 fatty acid intake, and this interaction was driven by the non-significant decrease in self-reported intakes in the placebo group (*p* = 0.074). While the interaction approached statistical significance for the dietary omega-6/omega-3 ratio (*p* = 0.051), *post-hoc* analysis indicated that this metric decreased in the MV group (*p* < 0.001), and MV values at POST were significantly less than placebo values at POST (*p* = 0.001). Notably, there were no interactions observed for data in Table 5 when these data were analyzed without the MV omega-3 and micronutrient values being added to the MV group (*data not shown*).

**Table 5:** Self-reported food intake.

**Self-reported intakes (units)**	**Time**	**Placebo (*n* = 46)**	**MV (*n* = 41)**	**Statistics**
Kcal (per day)	PRE	1,728 ± 581	1,706 ± 598	Time *p* = 0.304
	POST	1,650 ± 536	1,661 ± 567	G × T *p* = 0.780
CHO (g/day)	PRE	193 ± 82	209 ± 77	Time *p* = 0.387
	POST	186 ± 79	200 ± 82	G × T *p* = 0.885
Fiber (g/day)	PRE	19 ± 9	19 ± 11	Time *p* = 0.249
	POST	18 ± 7	19 ± 9	G × T *p* = 0.659
Protein (g/day)	PRE	77 ± 31	69 ± 28	Time *p* = 0.552
	POST	75 ± 26	67 ± 27	G × T *p* = 0.931
Fat (g/day)	PRE	75 ± 27	71 ± 32	Time *p* = 0.139
	POST	70 ± 24	66 ± 27	G × T *p* = 0.986
Omega-3 (g/day)	PRE	0.63 ± 0.64	0.41 ± 0.34	Time *p* = 0.047
	POST	0.57 ± 0.71	0.80 ± 0.46^*^	G × T *p* = 0.008
Omega-6 (g/day)	PRE	5.0 ± 3.2	4.1 ± 3.2	Time *p* = 0.998
	POST	3.9 ± 2.9	5.1 ± 3.4	G × T *p* = 0.029
Omega-6/omega-3 (ratio)	PRE	10.5 ± 7.2	10.1 ± 4.9	Time *p* = 0.014
	POST	10.1 ± 5.7	6.4 ± 4.0^*^^,^^#^	G × T *p* = 0.051
Saturated fat (g/day)	PRE	22 ± 7	21 ± 11	Time *p* = 0.559
	POST	22 ± 9	20 ± 9	G × T *p* = 0.987
Cholesterol (mg/day)	PRE	294 ± 187	216 ± 144	Time *p* = 0.459
	POST	254 ± 121	231 ± 142	G × T *p* = 0.110
Folate intake (μg/day)	PRE	208 ± 170	204 ± 166	Time *p* < 0.001
	POST	195 ± 150	786 ± 133^*^^,^^#^	G × T *p* < 0.001
Iron intake (mg/day)	PRE	9.8 ± 4.1	10.5 ± 5.7	Time *p* < 0.001
	POST	9.1 ± 3.9	19.0 ± 6.7^*^^,^^#^	G × T *p* < 0.001
Magnesium (mg/day)	PRE	137 ± 96	121 ± 94	Time *p* = 0.387
	POST	117 ± 77	184 ± 92^*^^,^^#^	G × T *p* = 0.262
Vitamin B12 (μg/day)	PRE	2.35 ± 2.24	1.47 ± 1.62	Time *p* < 0.001
	POST	1.64 ± 1.58	9.48 ± 1.14^*^^,^^#^	G × T *p* < 0.001
Vitamin D (μg/day)	PRE	1.8 ± 2.4	1.7 ± 2.1	Time *p* < 0.001
	POST	1.3 ± 1.5	51.2 ± 1.4^*^^,^^#^	G × T *p* < 0.001
Vitamin E (mg/day)	PRE	4.6 ± 4.2	4.5 ± 4.9	Time *p* < 0.001
	POST	3.9 ± 3.0	13.5 ± 3.6^*^^,^^#^	G × T *p* < 0.001
Vitamin K (μg/day)	PRE	105 ± 163	80 ± 165	Time *p* = 0.069
	POST	93 ± 148	148 ± 95^*^^,^^#^	G × T *p* = 0.009

Self-reported macronutrient and micronutrient intakes when taking into consideration the MV omega-3 and micronutrient values added to the MV group. All data presented as mean ± standard deviation values. Self-reported intakes that changed from PRE to POST in the MV group are indicated by “*” (p < 0.05). Self-reported intakes that were different in the MV vs. placebo group at POST are indicated by “#” (p < 0.05). While none of the variables were normally distributed, non-parametric statistics confirmed results provided by parametric statistics.

## Discussion

We sought to examine if a novel multivitamin supplement, which also contained omega-3 fatty acids, affected select serum micronutrient levels and red blood cell fatty acid profiles in pre-menopausal women during a 12-week supplementation period. Main findings from this study were that MV supplementation: (i) increased serum vitamin D levels and serum folate levels, and (ii) increased red blood cell omega-3 fatty acid content and altered various red blood cell fatty acid ratios suggestive of increased n-3 fatty acids and decreased n-6 fatty acid levels. We also observed an interaction for serum HDL cholesterol levels, and this was driven by the non-significant increase in this variable in the MV group. MV supplementation was safe and well-tolerated as determined by the blood pressure data, other serum markers (i.e., markers of liver and kidney function), and self-reported side effects. These findings are discussed in greater detail below.

NHANES data suggest millions of Americans have nutrient inadequacy or micronutrient deficient. Our finding that MV supplementation increased serum vitamin D levels compared to the placebo condition is a compelling finding. First, these data are impactful given that vitamin D deficiencies are highly prevalent (18). As shown in the results, it is also notable that several females in the MV group who were classified as vitamin D inadequate (<40 ng/dl) did not present low values at the POST time point. Thus, not only does MV supplementation increased serum vitamin D levels in general, but MV was effective at elevating serum vitamin D levels in females that presented a vitamin D inadequacy. Vitamin D deficiencies have been linked to endocrine disruption (19), depression (20), and the risk of developing osteoporosis later in life (21). While none of these physiological aspects were interrogated herein, future research is needed to determine if MV supplementation can mitigate these factors in individuals with low serum vitamin D levels. MV supplementation also increased serum folate levels. However, it should be noted that the serum folate data were confounded by the upper limit of the assay (i.e., 24.8 ng/ml). Another limitation is the lack of red blood cell folate data. The red blood cell folate assay has historically been recommended as a more reliable indicator of folate stores compared to the serum folate assay, as it is not affected by recent ingestion of food. Future research is needed to apply more precise assays capturing contribution of folate supplementation on red blood cell folate as well as folate metabolites.

As also mentioned previously, the dietary intake of omega-3 fatty acids, particularly long chain fatty acids, is generally low, and low omega-3 fatty acid intakes have been associated with diseases linked to low-grade inflammation (e.g., cardiovascular diseases, osteoarthritis, and cancer) (22). Not only did MV supplementation increase red blood cell omega-3 content, but it also reduced red blood cell arachidonic acid levels. This is an intriguing finding given that arachidonic acid is a substrate for pro-inflammatory prostaglandins (23). In lieu of these findings, however, it should be noted that our participant population was made up of relatively younger female adults who were apparently healthy. It is certainly possible that certain biomarkers reflective of systemic inflammation may have been marginally improved with MV supplementation given the supporting evidence cited herein. However, we opted not to examine how MV supplementation affected these markers (e.g., cytokines or prostaglandins) since prior data from our laboratory has shown these markers are low and difficult to detect in younger and apparently healthy participants (24). Notwithstanding, our data warrant examining the effects of MV supplementation in older persons who are susceptible to low-grade inflammation. It is also notable that the non-significant increase in serum HDL levels in MV vs. placebo participants may have been due to the omega-3 content in the MV supplement. In this regard, previous research has shown that lower-dose omega-3 supplementation (~300–400 mg/day) increases serum HDL cholesterol and positively affects serum lipid levels in healthy individuals (25, 26). Again, we are unsure as to whether this change in the MV-supplemented participants was meaningful given that they were relatively healthy. Nevertheless, and as alluded to above, these findings warrant longer-term MV supplementation studies in different population cohorts which may possess lower HDL levels and/or dyslipidemia.

## Limitations

A limitation of these current data is that this study was performed in relatively healthy, younger females. Thus, as mentioned above, it remains to be determined as to whether MV can mitigate various metabolic conditions that may be partially attributed to micronutrient deficiencies. As mentioned prior, our folate assay was confounded by the upper limit of the assay being reached by several individuals in the MV group, and this is an unresolved limitation. Another limitation is the lack of control for physical activity as well as sunlight exposure, as both can likely alter certain dependent variables (e.g., blood vitamin D and lipid levels). Finally, while MV supplementation reversed vitamin D inadequacies to a large degree, we are uncertain as to whether changes in other assayed variables yielded clinically meaningful physiological changes given that the participants were apparently healthy. Hence, as stated above, examining whether the MV supplement can mitigate nutritional deficiencies or metabolic perturbations is warranted.

## Conclusions

This study demonstrates that MV supplementation was effective at increasing serum vitamin D and folate levels, and favorably altering red blood cell fatty acid levels in healthy pre-menopausal women. MV supplementation appeared to be safe and well tolerated given that blood pressure and serum markers of clinical safety were not affected. All of these findings warrant future longer-term investigations as to whether MV supplementation can positively affect blood biomarkers and associated health outcomes in populations who are dyslipidemic, present low-grade inflammation, and/or are vitamin D or folate-deficient.

## Data Availability Statement

The raw data supporting the conclusions of this article will be made available by the authors, without undue reservation.

## Ethics Statement

The studies involving human participants were reviewed and approved by Auburn University Institutional Review Board. The patients/participants provided their written informed consent to participate in this study.

## Author Contributions

MR, KY, RA, NA, LB, and MS conceived and designed research. MR analyzed data and prepared figures. MR and SO drafted manuscript. All Auburn University co-authors (SO, PR, JM, JS, RA, KY, and MD) assisted with data collection and performed experiments. All authors interpreted results of experiments, edited and revised manuscript, and approved final version of manuscript.

## Conflict of Interest

NA, LB, AV, and MS are employed by Ritual. The remaining authors declare that the research was conducted in the absence of any commercial or financial relationships that could be construed as a conflict of interest. The authors declare that this study received funding from Ritual. The funder had the following involvement in the study: a) providing expertise in the area of omega-3 and multivitamin/mineral research, b) working with the Auburn investigators on designing an appropriate study. The funder was not involved with collection, analysis, interpretation of data, the writing of this article, or the decision to submit it for publication.

## References

[1] Bird JK Murphy RA Ciappio ED McBurney MI. Risk of deficiency in multiple concurrent micronutrients in children and adults in the United States. Nutrients. (2017) 9:655. 10.3390/nu9070655 28672791 PMC5537775

[2] USDA ARS. Usual Nutrient Intake from Food and Beverages, by Gender and Age, What We Eat in America, NHANES 2013-2016 (2019).

[3] Zhuang P Wang W Wang J Zhang Y Jiao J. Polyunsaturated fatty acids intake, omega-6/omega-3 ratio and mortality: findings from two independent nationwide cohorts. Clin Nutr. (2019) 38:848–55. 10.1016/j.clnu.2018.02.019 29551407

[4] Simopoulos AP. The importance of the ratio of omega-6/omega-3 essential fatty acids. Biomed Pharmacother. (2002) 56:365–79. 10.1016/S0753-3322(02)00253-6 12442909

[5] Romagnoli E., Mascia, M. L., Cipriani, C., Fassino, V., Mazzei, F., D'erasmo, E., et al. (2008). Short and long-term variations in serum calciotropic hormones after a single very large dose of ergocalciferol (vitamin D2) or cholecalciferol (vitamin D3) in the elderly. J Clin Endocrinol Metab. 93, 3015–3020. 10.1210/jc.2008-0350 18492750

[6] Thacher TD Obadofin MO O'brien KO Abrams SA. The effect of vitamin D2 and vitamin D3 on intestinal calcium absorption in Nigerian children with rickets. J Clin Endocrinol Metab. (2009). 94:3314–21. 10.1210/jc.2009-0018 19567516 PMC2741710

[7] Binkley N Gemar D Engelke J Gangnon R Ramamurthy R Krueger D. Evaluation of ergocalciferol or cholecalciferol dosing, 1,600 IU daily or 50,000 IU monthly in older adults. J Clin Endocrinol Metab. (2011) 96:981–8. 10.1210/jc.2010-0015 21289249 PMC3417158

[8] Heaney RP Recker RR Grote J Horst RL Armas LA. Vitamin D(3) is more potent than vitamin D(2) in humans. J Clin Endocrinol Metab. (2011) 96:E447–452. 10.1210/jc.2010-2230 21177785

[9] Lehmann U Hirche F Stangl GI Hinz K Westphal S Dierkes J. Bioavailability of vitamin D(2) and D(3) in healthy volunteers, a randomized placebo-controlled trial. J Clin Endocrinol Metab. (2013) 98:4339–45. 10.1210/jc.2012-4287 24001747

[10] Kiecolt-Glaser JK Belury MA Andridge R Malarkey WB Glaser R. Omega-3 supplementation lowers inflammation and anxiety in medical students: a randomized controlled trial. Brain Behav Immun. (2011) 25:1725–34. 10.1016/j.bbi.2011.07.229 21784145 PMC3191260

[11] Tartibian B Hajizadeh Maleki B Kanaley J Sadeghi K. Long-term aerobic exercise and omega-3 supplementation modulate osteoporosis through inflammatory mechanisms in post-menopausal women: a randomized, repeated measures study. Nutr Metab (Lond). (2011) 8:71. 10.1186/1743-7075-8-71 21999620 PMC3212907

[12] Kiecolt-Glaser JK Belury MA Andridge R Malarkey WB Hwang BS Glaser R. Omega-3 supplementation lowers inflammation in healthy middle-aged and older adults: a randomized controlled trial. Brain Behav Immun. (2012) 26:988–95. 10.1016/j.bbi.2012.05.011 22640930 PMC3398219

[13] Andrews KW Roseland JM Gusev PA Palachuvattil J Dang PT Savarala S. Analytical ingredient content and variability of adult multivitamin/mineral products: national estimates for the Dietary Supplement Ingredient Database. Am J Clin Nutr. (2017) 105:526–39. 10.3945/ajcn.116.134544 27974309 PMC5267296

[14] Kephart WC Wachs TD Thompson RM Brooks Mobley C Fox CD Mcdonald JR. Ten weeks of branched-chain amino acid supplementation improves select performance and immunological variables in trained cyclists. Amino Acids. (2016) 48:779–89. 10.1007/s00726-015-2125-8 26553453

[15] Roberson PA Romero MA Mumford PW Osburn SC Haun CT Vann CG. Protein supplementation throughout 10 weeks of progressive run training is not beneficial for time trial improvement. Front Nutr. (2018) 5:97. 10.3389/fnut.2018.00097 30456213 PMC6230989

[16] Lips P. Vitamin D deficiency and secondary hyperparathyroidism in the elderly: consequences for bone loss and fractures and therapeutic implications. Endocr Rev. (2001) 22:477–501. 10.1210/edrv.22.4.0437 11493580

[17] Cannell JJ Hollis BW Zasloff M Heaney RP. Diagnosis and treatment of vitamin D deficiency. Expert Opin Pharmacother. (2008) 9:107–18. 10.1517/14656566.9.1.107 18076342

[18] Roth DE Abrams SA Aloia J Bergeron G Bourassa MW Brown KH. Global prevalence and disease burden of vitamin D deficiency: a roadmap for action in low- and middle-income countries. Ann N Y Acad Sci. (2018) 1430:44–79. 10.1111/nyas.13968 30225965 PMC7309365

[19] Alpert PT Shaikh U. The effects of vitamin D deficiency and insufficiency on the endocrine and paracrine systems. Biol Res Nurs. (2007) 9:117–29. 10.1177/1099800407308057 17909164

[20] Anglin RE Samaan Z Walter SD McDonald SD. Vitamin D deficiency and depression in adults: systematic review and meta-analysis. Br J Psychiatry. (2013) 202:100–7. 10.1192/bjp.bp.111.106666 23377209

[21] Van Schoor NM Visser M Pluijm SM Kuchuk N Smit JH Lips P. Vitamin D deficiency as a risk factor for osteoporotic fractures. Bone. (2008) 42:260–6. 10.1016/j.bone.2007.11.002 18289505

[22] Deckelbaum RJ Torrejon C. The omega-3 fatty acid nutritional landscape: health benefits and sources. J Nutr. (2012). 142:587S−91S. 10.3945/jn.111.148080 22323763 PMC3278270

[23] Innes JK Calder PC. Omega-6 fatty acids and inflammation. Prostagland Leukot Essent Fatty Acids. (2018) 132:41–8. 10.1016/j.plefa.2018.03.004 29610056

[24] Mumford PW Kephart WC Romero MA Haun CT Mobley CB Osburn SC. Effect of 1-week betalain-rich beetroot concentrate supplementation on cycling performance and select physiological parameters. Eur J Appl Physiol. (2018) 118:2465–76. 10.1007/s00421-018-3973-1 30155761

[25] Franceschini G Calabresi L Maderna P Galli C Gianfranceschi G Sirtori CR. Omega-3 fatty acids selectively raise high-density lipoprotein 2 levels in healthy volunteers. Metabolism. (1991) 40:1283–6. 10.1016/0026-0495(91)90029-V 1961121

[26] Visioli F Rise P Plasmati E Pazzucconi F Sirtori CR Galli C. Very low intakes of N-3 fatty acids incorporated into bovine milk reduce plasma triacylglycerol and increase HDL-cholesterol concentrations in healthy subjects. Pharmacol Res. (2000) 41:571–6. 10.1006/phrs.1999.0650 10753557

